# Anterior Cruciate Ligament Reconstruction Using the Double Insertion of Semitendinosus and Gracilis Tendons (DIDT) Hamstring Technique: A Retrospective Study of 50 Cases at the Military Hospital of Nouakchott

**DOI:** 10.7759/cureus.96405

**Published:** 2025-11-09

**Authors:** Baba EL Bekay, Abdellahi Abed, Abderrahman Bebekar, Mohamed Mahmoud EL Hacen

**Affiliations:** 1 Department of Orthopedic and Trauma Surgery, Military Hospital of Nouakchott, Faculty of Medicine, Pharmacy and Odontostomatology, University of Nouakchott, Nouakchott, MRT; 2 Department of Radiology, Centre Hospitalier des Spécialités, Faculty of Medicine, Pharmacy and Odontostomatology, Nouakchott, MRT; 3 Department of Family Medicine, Cheikh Zayed Hospital, Nouakchott, MRT

**Keywords:** anterior cruciate ligament, arthroscopy, hamstring autograft, lysholm score, mauritania, tegner scale

## Abstract

Background: Anterior cruciate ligament (ACL) rupture is a frequent cause of knee instability in young and active individuals. Reconstruction using hamstring tendon autografts offers good biomechanical stability and minimizes donor site morbidity. This study aimed to evaluate the clinical and functional outcomes of ACL reconstruction performed in our institution using the double-bundle hamstring technique.

Methods: This retrospective study included 50 patients who underwent arthroscopic ACL reconstruction using the double insertion of semitendinosus and gracilis tendons (DIDT) technique at the Military Hospital of Nouakchott, Mauritania, between January 2019 and December 2023. Demographic data, injury mechanisms, surgical details, complications, and functional outcomes were analyzed. Functional results were assessed using the Lysholm and Tegner activity scores with a minimum follow-up of 12 months.

Results: The mean age was 29.3 years (range: 21-39 years), with a male predominance (90%). Sports trauma, mainly football, accounted for 60% of injuries. At final follow-up, 80% of patients achieved good or excellent functional results according to the Lysholm and Tegner scales. Postoperative complications included two superficial infections and one case of Volkmann’s ischemic contracture. MRI confirmed graft integrity in 96% of patients. The average time to return to sports was eight months.

Conclusion: ACL reconstruction using the hamstring tendon autograft provides excellent functional recovery, high patient satisfaction, and low complication rates. This technique is particularly suitable for resource-limited settings because of its simplicity, reproducibility, and favorable outcomes.

## Introduction

The anterior cruciate ligament (ACL) plays a crucial role in maintaining anteroposterior and rotational stability of the knee joint. Its rupture is one of the most common ligamentous injuries, particularly among young and physically active individuals, often resulting in significant functional impairment and prolonged interruption of sporting activities [1-3]. If left untreated, ACL deficiency may lead to recurrent instability, secondary meniscal lesions, and early degenerative changes of the knee joint [4].

Over the past three decades, arthroscopic ACL reconstruction has evolved substantially, with several graft options available. Among these, the hamstring tendon autograft using the double insertion of semitendinosus and gracilis tendons (DIDT) technique has become widely adopted because of its minimal donor-site morbidity, reduced anterior knee pain, and favorable biomechanical performance compared with bone-patellar tendon-bone (BPTB) grafts [5-8].

In resource-limited environments, where access to advanced instrumentation, specialized implants, and structured rehabilitation programs may be restricted, the DIDT technique provides a practical balance between technical feasibility, cost-effectiveness, and functional efficiency [9]. However, few studies from sub-Saharan Africa have reported outcomes of this technique, even though differences in patient characteristics, equipment availability, and postoperative rehabilitation can significantly affect results [9].

In our context, where access to MRI and absorbable fixation materials is available only in a few tertiary hospitals, documenting outcomes of ACL reconstruction using hamstring autografts provides valuable data for surgeons practicing in similar healthcare environments.

Therefore, the aim of this study was to evaluate the clinical and functional outcomes of ACL reconstruction using hamstring tendon autografts, assessed at 12 months postoperatively with the International Knee Documentation Committee (IKDC) [10], Lysholm [11] scores, and to compare these results with data previously reported in the literature.

## Materials and methods

Study design and setting

This retrospective descriptive study was conducted in the Department of Traumatology and Orthopedics at the Military Hospital of Nouakchott, Mauritania. It included patients who underwent arthroscopic ACL reconstruction between January 2019 and December 2023. The study protocol complied with institutional ethical standards, and all patients provided written informed consent prior to surgery and data inclusion.

Study population

A total of 50 patients presenting with chronic knee instability secondary to ACL rupture were included. Data were collected from operative reports, hospital archives, and outpatient follow-up records. All procedures were performed by a single experienced orthopedic surgeon to ensure consistency and minimize inter-operator variability. The surgical technique used was the DIDT autograft.

Inclusion and exclusion criteria

Inclusion criteria were as follows: chronic knee instability confirmed by clinical examination and magnetic resonance imaging (MRI); primary ACL rupture treated surgically using the DIDT technique; and a minimum follow-up period of 12 months.

Exclusion criteria were as follows: ACL reconstruction using other graft types or techniques; associated fractures or multi-ligament injuries; and incomplete or non-exploitable medical records.

Surgical technique

All surgeries were performed under spinal anesthesia, with the patient in the supine position, the knee flexed to 90°, and a pneumatic tourniquet applied to the thigh. Standard anteromedial and anterolateral arthroscopic portals were created. The arthroscopic system and fixation materials used were Karl Storz® (Tuttlingen, Germany) equipment and bioabsorbable interference screws (Karl Storz®).

The semitendinosus and gracilis tendons were harvested through a small anteromedial incision and prepared to form a four-strand graft with a minimum length of 7 cm. The femoral tunnel was drilled through the anteromedial portal at the 10:30 o’clock position for right knees and 1:30 o’clock for left knees, using a tibial guide set at 55°. Associated procedures, such as meniscal repair or partial meniscectomy, were performed when indicated.

A Redon drain was left in place for 24 hours postoperatively, and a knee brace was applied in extension. All patients received perioperative antibiotic prophylaxis (cefazolin 2 g IV, 30 minutes before incision, continued for 24 hours), thromboprophylaxis, and multimodal analgesia.

The DIDT technique is associated with minimal donor-site morbidity, thus reducing anterior knee pain and extension weakness compared with BPTB grafts [8].

Postoperative rehabilitation

All patients followed a standardized rehabilitation protocol supervised by a physiotherapy team. Passive range-of-motion exercises and non-weight-bearing ambulation began on postoperative day one. Partial weight-bearing was authorized after three weeks, and full weight-bearing with muscle strengthening was introduced progressively after six weeks. The goal was to achieve full flexion by day 45. Return to sports was generally permitted after eight months, depending on functional recovery and knee stability.

Postoperative follow-up and evaluation

Follow-up was conducted by an independent orthopedic surgeon at three, six, and 12 months postoperatively. Functional outcomes were assessed using the IKDC [10] and Lysholm [11] scores, both validated tools for ACL reconstruction evaluation [9,10]. Missing data were managed by excluding incomplete records from the outcome analysis.

Data collection and statistical analysis

Data collection included demographic variables (age, sex, and occupation), injury mechanism, surgical details, intraoperative and postoperative complications, and functional outcomes.

Statistical analysis was performed using Microsoft Excel 2016 (Microsoft Corp., Armonk, NY). Continuous variables were expressed as means and standard deviations, and categorical variables as frequencies and percentages.

Ethical considerations

The study was conducted in accordance with the Declaration of Helsinki, and ethical approval was obtained from the institutional review board of the Military Hospital of Nouakchott (approval number: MHN/IRB/ACL/2024/05).

## Results

Epidemiological findings

The study included 50 patients with ACL rupture. The mean age was 29.3 years (range: 21-39 years). The most affected age group was 25-30 years, accounting for 19 patients (38%), followed by those under 25 years (12 patients, 24%), 30-35 years (11 patients, 22%), and over 35 years (eight patients, 16%) (Table 1).

**Table 1:** Distribution of patients according to age

Age group	Number of patients	Percentage (%)
<25 years	12	24
25–30 years	19	38
30–35 years	11	22
>35 years	8	16

There was a marked male predominance with 45 men (90%) and five women (10%), corresponding to a sex ratio of 9:1. The right knee was involved in 32 cases (64%), while the left knee was affected in 18 cases (36%).

Occupational and sports activity

**Figure 1:**
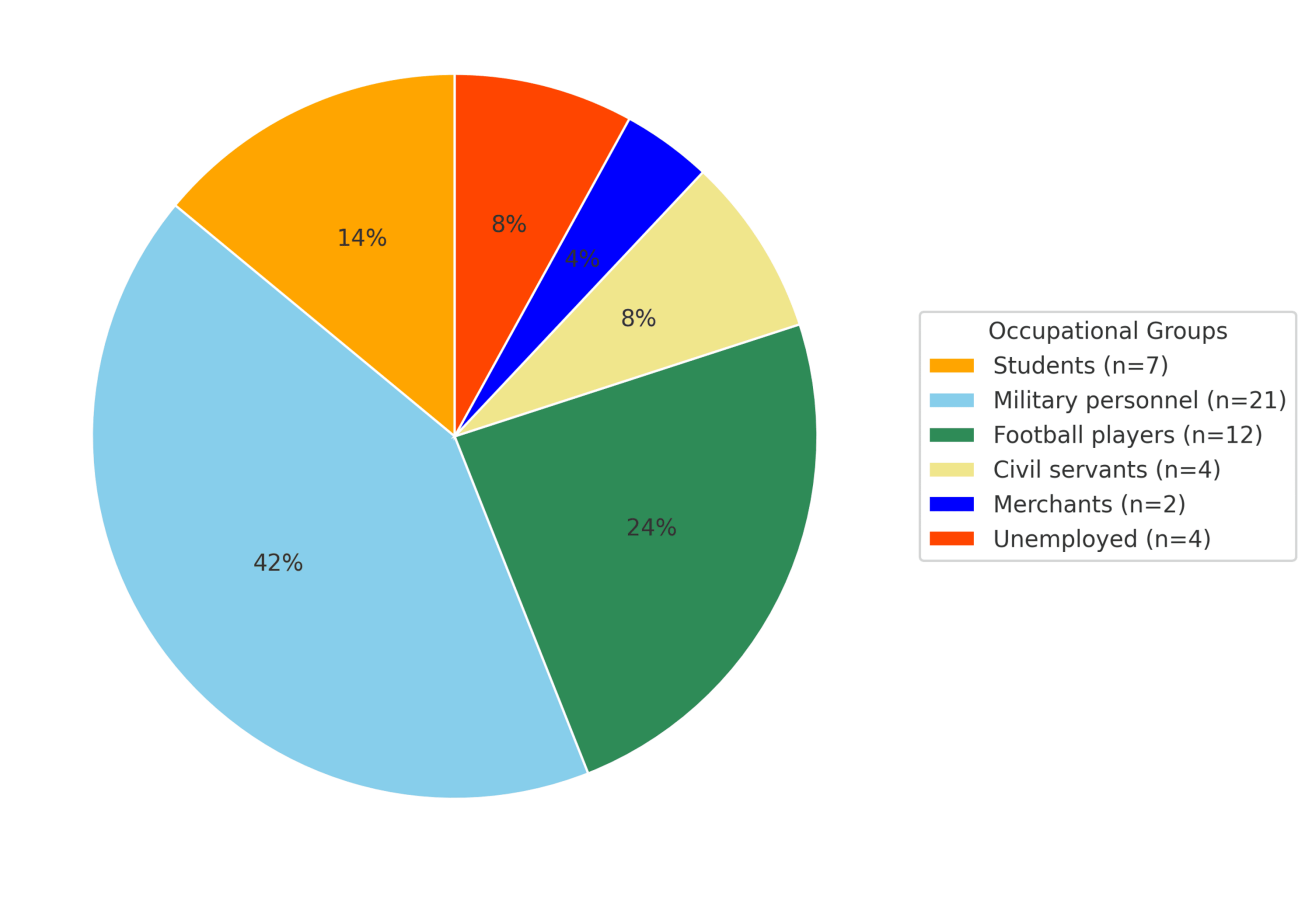
Distribution of patients according to their occupation

**Figure 2:**
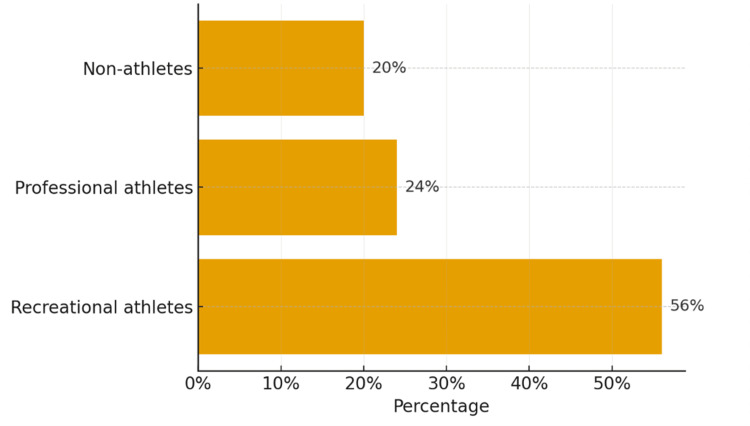
Distribution of patients according to their sports activity level

Among the 50 patients, 21 (42%) were military personnel, 12 (24%) were professional football players, seven (14%) were students, four (8%) were civil servants, two (4%) were merchants, and four (8%) were unemployed (Figure 1). Regarding physical activity, 28 patients (56%) practiced recreational sports, 12 (24%) were professional athletes, and 10 (20%) reported no sports activity (Figure 2).

Etiology and mechanism of injury

**Figure 3:**
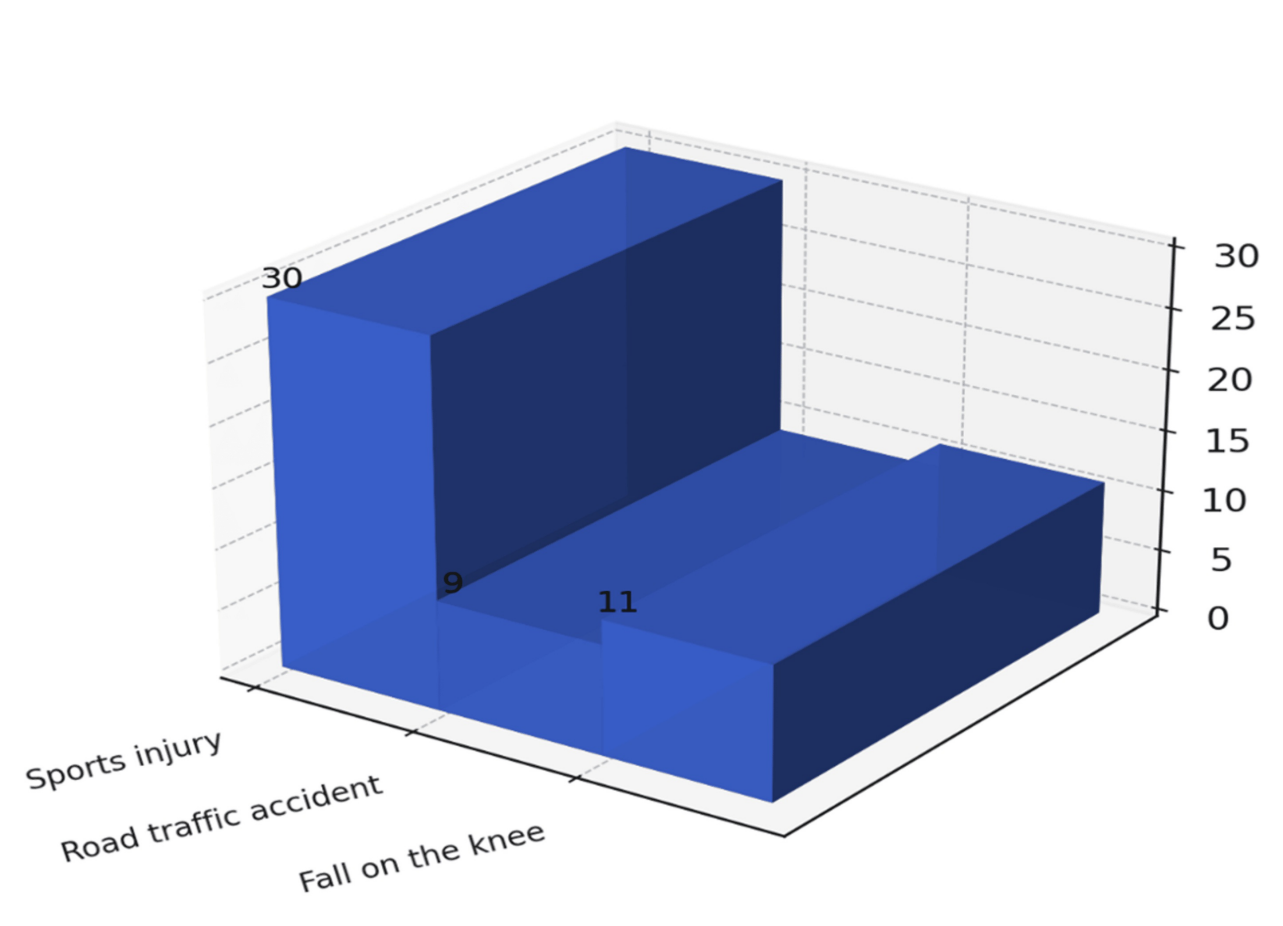
Etiology of anterior cruciate ligament ruptures in the study population

Sports trauma was the main cause of ACL rupture, observed in 30 patients (60%), followed by road traffic accidents (nine patients, 18%) and domestic falls (11 patients, 22%) (Figure 3). The most frequent injury mechanism was valgus-flexion-external rotation in 35 cases (70%), followed by varus-flexion-internal rotation in 15 cases (30%). Among the 30 football-related injuries, 25 were caused by sudden “on-field stop” movements.

Delay between trauma and surgery

**Figure 4:**
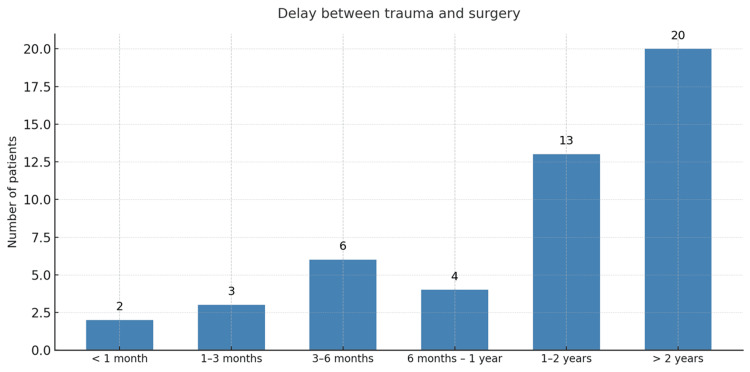
The distribution of patients according to the time between trauma and surgery

The interval between trauma and surgical reconstruction ranged from six months to 48 months, with a mean of 25 months (≈2 years) (Figure 4).

At presentation, 12 patients (24%) had acute knee trauma characterized by pain, swelling, and complete functional impairment. Chronic knee instability was reported in 38 patients (76%), among whom 14 (28%) experienced locking and nine (18%) reported pain during deep flexion or squatting.

The Lachman and anterior drawer tests were positive in 38 patients (76%), while the pivot-shift test was positive in 30 patients (60%). Clinical examination was difficult in 12 patients (24%) due to marked pain, joint effusion, and limited range of motion at presentation (Table 2).

**Table 2:** Results of ligamentous clinical tests

Test type	Number of cases	Percentage (%)
Lachman test	38	76
Anterior drawer	38	76
Pivot-shift	30	60
Difficult examination	12	24

Joint effusion was noted in 10 cases (20%), a positive grinding test suggesting meniscal injury in 23 cases (46%), and quadriceps atrophy in 38 cases (76%).

Radiological findings

Standard knee radiographs were normal in all cases (100%). MRI confirmed the diagnosis of ACL rupture in all patients: 39 (78%) had complete rupture, six (12%) had partial rupture, and five (10%) had suspected but unconfirmed lesions.

The topographic distribution revealed 16 mid-substance ruptures, 23 tibial or femoral detachments, and five instances of complete absence of visualization. Associated lesions included lateral meniscus tears in 16 cases (32%), medial meniscus tears in seven cases (14%), and osteochondral lesions in 17 cases (34%), mainly on the femoral condyles.

Surgical technique and postoperative care

All patients underwent arthroscopic ACL reconstruction using a DIDT autograft. Fixation was performed with resorbable interference screws. Meniscal repair was performed in four cases, regularization in six, and partial meniscectomy in 12.

All patients received antibiotic prophylaxis, thromboprophylaxis, and multimodal analgesia. The average hospital stay was 24 hours.

Complications

Intraoperative complications included six cases (12%) of graft jamming and six cases (12%) of femoral screw fracture. Fixation was revised in four cases and deemed stable in two. These events were attributed to the learning curve associated with arthroscopic tunnel placement and screw insertion.

Postoperatively, one patient (2%) developed Volkmann’s ischemic contracture secondary to necrotizing fasciitis, progressing to irreversible ischemia and resulting in below-knee amputation. This severe event was attributed to an acute postoperative compartment syndrome, rather than the reconstruction technique itself.

Two patients (4%) presented with purulent discharge and localized edema at the incision site, raising suspicion of possible intra-articular extension. Arthroscopic lavage was performed in both cases for diagnostic confirmation and local debridement, revealing no joint contamination. Both patients recovered fully after lavage and antibiotic therapy.

No thromboembolic events were observed.

Functional outcomes

At the final follow-up (12 months), 48 patients (96%) demonstrated negative Lachman, anterior drawer, and pivot-shift tests. MRI confirmed graft integrity in 48 patients (96%), while two patients (4%) had graft failure (one femoral detachment and one elongation) and underwent revision surgery elsewhere.

Residual knee pain was reported by two patients (4%), and four (8%) experienced mild instability during intense activity. Four patients (8%) developed stiffness secondary to delayed rehabilitation; two recovered after prolonged physiotherapy, one required arthrolysis, and one retained flexion limited to 90°.

Return to activity and functional scores

Return to work occurred after a mean of 2.5 months (range: three to six months). The mean time to return to sports was eight months, with military personnel resuming physical activities after approximately seven months. All 12 professional football players returned to competition without complications.

According to the Lysholm [11] and Tegner [12] scales, 40 patients (80%) achieved good or excellent outcomes, nine (18%) were rated fair, and one (2%) was rated poor, mainly due to residual pain or limited flexion.

## Discussion

Epidemiological characteristics

The mean age of our patients (29.3 years) aligns with previous reports by Hajjioui (30 years) [1] and Giraud et al. (27.6 years) [2], confirming that ACL rupture predominantly affects young, physically active individuals. The marked male predominance (90%) is consistent with studies by Lerat et al. (80.4%) [3], Elhassib (96%) [4], and Mall et al. [5], reflecting the higher exposure of men to pivoting sports and military activities.

The right knee was most commonly affected (64%), similar to Elhassib's study [4], whereas Hajjioui [1] reported left-sided predominance (69%). The high proportion of military personnel (42%) and football players (24%) reflects the demanding physical nature of these occupations, consistent with Laffargue et al. [7] and Lerat et al. [3], who identified pivoting sports as leading causes of ACL rupture.

Mechanism and etiology of injury

Sports trauma was the predominant cause (60%), in agreement with studies by Lerat et al. [3], Elhassib [4], and Gaudot et al. [6]. The valgus-flexion-external rotation mechanism was most frequent (70%), comparable to the 54% reported by Hajjioui [1]. These findings confirm that non-contact pivoting movements, particularly in football, remain the leading cause of ACL injury.

Delay between trauma and surgery

The mean delay between trauma and surgery was 25 months, comparable to the 18.3 months reported by Laffargue et al. [7]. This delay likely reflects diagnostic and financial constraints and limited access to surgical facilities, common in resource-limited environments. Such prolonged intervals may increase the risk of secondary meniscal and chondral damage, thereby influencing long-term outcomes.

Clinical and radiological findings

Knee instability was the predominant symptom (99%), followed by pain (28%), swelling (24%), and locking (18%), consistent with Elhassib's study [4]. The positivity rates of the Lachman (76%) and pivot-shift (60%) tests were comparable to those described by Boerre and Ackroyd [13].

The variability observed between clinical examination and imaging findings may be explained by the complex anatomical orientation and biomechanical structure of the ACL, as described by Arnoczky [14].

MRI was essential for diagnosis and for detecting associated meniscal and osteochondral lesions, consistent with Zaroual's study [15], confirming its role as the imaging gold standard in ACL injury assessment.

Surgical technique, learning curve, and rehabilitation

All patients underwent arthroscopic reconstruction using the DIDT autograft. This method allows accurate tunnel placement, good graft integration, and reduced donor-site morbidity, in agreement with El Khadime [16] and Gaudot et al. [6]. According to Plaweski et al. [8], hamstring autografts offer equivalent stability to BPTB grafts with fewer anterior knee complications.

Intraoperative complications such as graft jamming and screw fracture observed in our series reflect the learning curve typical of surgeons introducing arthroscopic ACL reconstruction in low-resource settings. With increased experience, better equipment handling, and standardized steps, these complications tend to decrease significantly.

Rehabilitation began early, focusing on gradual mobilization and quadriceps strengthening. The average rehabilitation period was three months, comparable to Zaroual's study [15]. Despite the absence of formal physiotherapy centers in some cases, early mobilization under supervision was essential for preventing stiffness and optimizing functional outcomes.

Complications

The overall complication rate in our study was low. The single case of Volkmann’s ischemic contracture leading to amputation was an exceptional event, attributed to an acute postoperative compartment syndrome rather than the reconstruction technique itself.

Two cases of purulent wound infection were successfully managed with arthroscopic lavage and antibiotics. Lavage was performed as a precautionary measure to exclude intra-articular contamination, and both patients recovered completely. These results compare favorably with Zaroual [15], who reported three cases of septic arthritis and four cases of persistent anterior knee pain, underscoring the effectiveness of rigorous asepsis and early intervention.

Functional outcomes

At the final follow-up, 96% of patients achieved stable knees with negative Lachman and pivot-shift tests. According to the Lysholm [11] and Tegner [12] scales, 80% had “good” or “excellent” outcomes, consistent with studies by Hajjioui [1] and Zaroual [15]. The average return to work and sport (2.5 and eight months, respectively) also aligns with previous studies [15,16].

A meta-analysis by Chen et al. [17] confirmed that hamstring autografts provide outcomes equivalent or superior to BPTB grafts, with lower anterior knee pain rates. These findings support the clinical effectiveness and safety of the DIDT technique, even in environments with limited resources.

Relevance in a resource-limited context

While ACL reconstruction using hamstring autografts is a well-established technique, its application in low-resource settings remains underreported. In our institution, access to MRI and absorbable fixation devices is available but limited, and physiotherapy services are often constrained. Despite these challenges, satisfactory functional recovery was achieved, demonstrating that high-quality outcomes can be obtained with standard arthroscopic equipment and careful postoperative monitoring.

This experience supports the view that feasible and reproducible surgical solutions can be successfully implemented in sub-Saharan environments when proper patient selection, training, and structured rehabilitation protocols are followed [9,18].

Study limitations

This study presents several limitations. Its retrospective design and modest sample size limit statistical power and generalizability. The absence of a control group prevents direct comparison with other graft types or fixation systems. Furthermore, functional evaluation was limited to a one-year follow-up, which may not reflect long-term stability.

Nevertheless, our study provides meaningful regional data illustrating that ACL reconstruction with hamstring autografts is both feasible and effective in resource-limited settings.

## Conclusions

ACL rupture remains a major cause of knee instability and functional limitation among young, active individuals. In this series, arthroscopic ACL reconstruction using the DIDT technique achieved satisfactory clinical and functional outcomes, with a low complication rate and high patient satisfaction.

This technique offers an optimal balance between technical simplicity, cost-effectiveness, and biomechanical reliability, making it particularly suitable for resource-limited healthcare environments.

However, the results should be interpreted with caution, given the retrospective design, relatively small sample size, and lack of a control group. Further prospective, comparative studies with longer follow-up are needed to confirm these findings and strengthen the evidence base for ACL reconstruction practices in low-resource settings.
